# Association between coexisting physical, cognitive, and social frailty and sarcopenia in patients undergoing maintenance hemodialysis

**DOI:** 10.3389/fmed.2025.1692427

**Published:** 2025-10-23

**Authors:** Luchen Chen, Yanyu Fang, Yongze Dong, Mengjiao Zhao, Shiyan Yao, Qi Zhong, Liying Wang, Haixin Song, Guannan Ma, Huajuan Shen

**Affiliations:** ^1^College of Nursing, Zhejiang Chinese Medical University, Hangzhou, China; ^2^Department of Nephrology, Zhejiang Provincial People’s Hospital, Hangzhou, China; ^3^School of Public Health and Nursing, Hangzhou Normal University, Hangzhou, China; ^4^Department of Nursing, Zhejiang Provincial People’s Hospital, Hangzhou, China

**Keywords:** maintenance hemodialysis, frailty, sarcopenia, correlation, influencing factors analysis

## Abstract

**Objectives:**

To investigate the influencing factors of coexisting physical, cognitive, and social frailty in MHD patients and explore its association with sarcopenia, in order to provide a reference for developing comprehensive intervention strategies targeting multidimensional frailty.

**Methods:**

A convenience sample of MHD patients receiving treatment at the hemodialysis centers of two general hospitals in Hangzhou from July to August 2025 was enrolled. Data were collected using a general information questionnaire, Fried Frailty Phenotype Scale, Social Frailty Scale, Mini-Mental State Examination (MMSE), Modified Quantitative Subjective Global Assessment (MQSGA), Self-Rating Depression Scale (SDS), Social Support Rating Scale (SSRS), and International Physical Activity Questionnaire-Short Form (IPAQ-SF). Pearson correlation analysis and binary logistic regression were used to examine the co-occurrence of physical, cognitive, and social frailty and its association with sarcopenia.

**Results:**

A total of 336 questionnaires were distributed and 325 valid responses were collected. Among the 325 MHD patients, the proportions of individuals with frailty in 0, 1, 2, and 3 domains were 60.0% (*n* = 195), 15.4% (*n* = 50), 32.0% (*n* = 104), and 11.1% (*n* = 36), respectively. The overall prevalence of sarcopenia was 16.0% (*n* = 52), and a significant positive correlation was observed between sarcopenia and multidimensional frailty (*r* = 0.488, *P* < 0.001). Logistic regression analysis identified prealbumin level, social support, age, SGA score, and sarcopenia as significant predictors of coexisting physical, cognitive, and social frailty in MHD patients (*P* < 0.05). After controlling for potential confounders, sarcopenia remained significantly associated with multidimensional frailty (*P* < 0.05).

**Conclusion:**

Approximately 11.1% of MHD patients experience coexisting physical, cognitive, and social frailty. Sarcopenia is significantly associated with this multidimensional frailty. Early identification and intervention targeting sarcopenia and its related factors may help prevent or mitigate frailty in MHD patients.

## 1 Introduction

End-stage renal disease (ESRD) has emerged as a major global public health concern, with a prevalence of approximately 10%∼13% and a mortality rate reaching up to 30% (1). Maintenance hemodialysis (MHD) is the most commonly employed renal replacement therapy for patients with ESRD. Although it alleviates symptom burden and prolongs survival, the long-term dialysis process leads to substantial loss of protein-bound amino acids and proteins. This, compounded by patients’ sedentary or bed-bound behavior, further exacerbates impaired muscle synthesis and decline in physical performance, ultimately resulting in frailty (2). Studies have shown that the prevalence of frailty in MHD patients is 37.4%∼43.6% (3, 4), which is higher than that observed in elderly patients with atrial fibrillation (5) or hospitalized individuals with diabetes (6).

Frailty is a multidimensional and complex clinical syndrome encompassing four interrelated and overlapping domains: physical, cognitive, psychological, and social. It is characterized by a decline in physiological reserves or dysregulation across multiple systems, resulting in increased vulnerability to stressors and impaired homeostatic stability (7, 8). Physical frailty is a medical syndrome primarily characterized by declines in strength, endurance, and physiological function (9). Cognitive frailty refers to a decline in cognitive reserve and function in the absence of subjective memory complaints and without Alzheimer’s disease or other forms of dementia (10). Social frailty is defined as a state in which individuals experience deficiencies in social needs, support, resources, and satisfaction, accompanied by reduced self-management capacity across the lifespan (8, 11). The different domains of frailty interact through complex, bidirectional mechanisms (8, 12). Extensive research has demonstrated close interconnections among these domains (13, 14). Because the characteristics and underlying mechanisms of each domain overlap, improvement or deterioration in one domain often leads to corresponding changes in others. Recognizing and understanding these interrelationships is crucial for improving related health outcomes. Accumulating evidence indicates that multidimensional frailty is associated with increased risks of cardiovascular events and all-cause mortality (4, 15, 16). Compared to age-related physiological decline alone, factors such as sarcopenia, malnutrition, fatigue, and inadequate social support more readily contribute to multidimensional frailty in MHD patients (17, 18).

Sarcopenia, a progressive age-related loss of skeletal muscle mass and strength, is considered a precursor syndrome of frailty in MHD patients. It not only directly leads to reduced muscle mass and strength, resulting in physical functional decline, but also indirectly contributes to psychological depression and social isolation through mechanisms such as decreased mobility and reduced protein-energy metabolism. This forms a vicious cycle of “sarcopenia–frailty” (19–21).

However, current research on sarcopenia and frailty in MHD patients predominantly focuses on single or dual dimensions, with limited studies addressing the coexistence of multidimensional frailty and sarcopenia. The relationship between the two remains unclear. To date, only Yi et al. (22) have explored the association between sarcopenia and physical and cognitive frailty in MHD patients, yet the study lacked relevant clinical indicators, limiting its interventional applicability. Therefore, this study aims to investigate the prevalence and coexistence patterns of physical, psychological, and social frailty among patients undergoing maintenance hemodialysis, and to further examine both the independent and combined effects of these frailty dimensions on sarcopenia. The findings are expected to provide a scientific basis for the development of comprehensive and multidimensional frailty intervention strategies in clinical practice.

## 2 Materials and methods

### 2.1 Study design

A convenience sampling method was used to recruit patients undergoing maintenance hemodialysis (MHD) at the Hemodialysis Centers of two general hospitals in Hangzhou from July to August 2025. Inclusion criteria were: (1) age ≥ 18 years, and (2) receiving MHD for ≥ 3 months. Exclusion criteria included: (1) inability to cooperate with examinations or questionnaires; (2) contraindications to bioelectrical impedance analysis (e.g., cardiac pacemaker or metallic implants); (3) severe comorbidities such as malignancy or acute heart failure; (4) history of neurological disorders (e.g., Alzheimer’s disease, dementia, epilepsy, Parkinson’s syndrome); (5) pregnancy or lactation; (6) use of corticosteroids or immunosuppressants within the past six months.

Based on multivariate regression requirements, the minimum sample size should be 5–10 times the number of independent variables. With 44 variables included, the estimated sample size was 220–440. Considering a 20% potential dropout rate, the final required sample size was 264–528. Ultimately, 325 patients were enrolled.

### 2.2 Measurements

A questionnaire was used to collect demographic data, including age, sex, education level, residence, smoking, and alcohol consumption. key clinical and laboratory parameters-such as primary disease, comorbid diabetes, dialysis type, dialysis vintage, vascular access, blood urea nitrogen, serum creatinine, fasting glucose, electrolytes, albumin, hemoglobin, globulin, prealbumin, lipids, bilirubin, neutrophil-to-lymphocyte ratio, parathyroid hormone, vitamin D, pre-dialysis β_2_-microglobulin, high-sensitivity c-reactive protein, and kt/V-were verified via electronic medical records using the most recent pre-dialysis lab results. anthropometric and body composition measurements-including BMI, calf circumference, mid-upper arm circumference, mid-arm muscle circumference, extracellular water ratio, body cell mass index, body fat percentage, and phase angle-were assessed using a standardized body composition analyzer. all measurements were performed by trained personnel following standardized protocols to ensure consistency and minimize bias.

#### 2.2.1 Fried Frailty Phenotype

The Fried Frailty Phenotype, developed by Fried and Tangen (23) based on the cycle of frailty model, is one of the most widely used tools for frailty assessment in clinical practice (23). It includes five components: unintentional weight loss, slowed walking speed, decreased grip strength, reduced physical activity, and exhaustion. Each component is scored as 1 point if present. A total score of 0 indicates non-frailty, 1–2 indicates pre-frailty, and a score of 3 or more indicates frailty. This tool is simple to administer, has good predictive validity, and demonstrates good internal consistency, with a Cronbach’s alpha coefficient of 0.826.

#### 2.2.2 Mini-Mental State Examination (MMSE)

Mini-Mental State Examination, developed by Folstein et al. (24) is used to assess cognitive function across various domains, including orientation to time, attention and calculation, memory, and language. The total score ranges from 0 to 30, with higher scores indicating better cognitive function. The cut-off scores for normal cognitive function vary by education level: > 17 for illiterate individuals, > 20 for those with primary education, and > 24 for individuals with junior high school education or above. The Chinese version of the MMSE was translated and validated by Wang and Zhang (25), with a test-retest reliability coefficient of 0.91.

#### 2.2.3 Modified Quantitative Subjective Global Assessment (MOSGA)

Modified Quantitative Subjective Global Assessment comprises seven items and uses a five-point scale to evaluate an individual’s nutritional status. The assessed domains include weight change, dietary intake, functional capacity and physical activity, gastrointestinal symptoms, the relationship between disease and nutritional requirements, metabolic demand, and physical examination findings. Higher scores indicate poorer nutritional status. The total score ranges from 7 to 35, with a score of ≤ 10 indicating good nutritional status and > 10 indicating malnutrition.

#### 2.2.4 Self-Rating Depression Scale (SDS)

The SDS, developed by Zung (26), includes 20 items scored on a four-point scale, with 10 items being reverse-scored. The raw score is obtained by summing the scores of all items, which is then multiplied by 1.25 and rounded to the nearest whole number to yield the standard score. Standard scores below 53 indicate no depression; 53∼62 indicate mild depression; 63∼72 indicate moderate depression; and scores above 72 indicate severe depression. The Chinese version demonstrates good reliability and validity, with a Cronbach’s alpha coefficient of 0.725.

#### 2.2.5 International Physical Activity Questionnaire-Short Form (IPAQ-SF)

International Physical Activity Questionnaire-Short Form consists of seven items, including one item assessing daily sedentary time and six items evaluating the participant’s vigorous, moderate, and light physical activities during the past week. Only activities lasting at least 10 min are considered valid. Physical activity levels are quantified using Metabolic Equivalent of Task (MET) minutes per week, calculated as: MET value × frequency per week (days/week) × duration per day (minutes/day). The assigned MET values are 3.3 for light intensity, 4.0 for moderate intensity, and 8.0 for vigorous intensity activities. Total MET-minutes per week are categorized as follows: ≥ 3,000 indicates high physical activity level, 600–2,999 moderate level, and < 600 low level.

#### 2.2.6 Social Support Rating Scale (SSRS)

The SSRS, developed by Xiao (27), is designed to assess an individual’s social support status. The scale comprises three dimensions: subjective support, objective support, and support utilization, totaling 10 items. Items 1∼4 and 8∼10 are rated on a four-point Likert scale (1∼4). Item 5 includes 5 sub-items, each scored from 1 to 4. Items 6 and 7 are scored according to the number of sources of support. Total scores are categorized into three levels of social support: ≤ 22 indicates low support, 23∼44 moderate support, and 45∼66 high support. The original scale demonstrated strong reliability, with a Cronbach’s alpha coefficient of 0.896 and test-retest reliability between 0.89 and 0.94.

#### 2.2.7 Help, participation, loneliness, financial and talk scale (HALFT)

The HALFT, developed by Ma et al. (28), is primarily used to assess an individual’s social frailty status. It includes five components: whether the individual has helped others in the past year, participation in social activities, feelings of loneliness in the past week, whether the income in the past year was sufficient to meet basic living needs, and whether there are close friends or relatives to confide in. The total score ranges from 0 to 5, with a score of ≥ 3 indicating social frailty and < 3 indicating no social frailty. The scale demonstrated moderate internal consistency, with a Cronbach’s alpha coefficient of 0.602.

#### 2.2.8 Diagnostic criteria for sarcopenia

The diagnostic criteria recommended by the Asian Working Group for Sarcopenia 2019 Consensus Update (29) were adopted. The criteria include: ➀ Grip strength: > 28 kg for men, > 18 kg for women; ➁ 6 m walking speed: < 1.0 m/s; ➂ Appendicular skeletal muscle mass index (ASMI): < 7.0 kg/m^2^ for men, < 5.7 kg/m^2^ for women. A diagnosis of sarcopenia is made if criterion ➂ is met along with either criterion ➀ and/or criterion ➁.

### 2.3 Data collection and quality control

Before data collection, two researchers received standardized training. During the survey, they explained the study purpose and questionnaire procedures to patients and obtained informed consent before distribution. Some clinical data were retrieved from the hospital’s hemodialysis records. Grip strength was measured pre-dialysis using a dynamometer (AJW Technology Co., Germany). Patients stood naturally with arms relaxed and used the non-fistula hand (or dominant hand if catheterized). Two measurements were taken, and the maximum value was recorded. Gait speed was assessed over a 6 m walk at a normal pace without assistance. Two trials were conducted, and the average time was used.

Anthropometric measurements-including triceps skinfold thickness and mid-upper arm circumference-were performed 30–60 min post-dialysis, before eating or drinking. Appendicular skeletal muscle mass was measured with the InBody S10 body composition analyzer. Patients removed shoes, socks, and all metal objects, lay supine with arms abducted, and remained still until the device beeped to signal completion. BMI and mid-upper arm muscle circumference were calculated independently by the researchers.

A total of 336 questionnaires were distributed, of which 325 were valid, yielding an effective response rate of 96.7%.

### 2.4 Ethical considerations

This study was approved by the Ethics Committee of Zhejiang Provincial People’s Hospital [Approval No. 2025 (221)] and the Ethics Committee of Lin’an First People’s Hospital of Hangzhou [Approval No. 2025 (08)]. The questionnaire was used with authorization from the original developer. Written informed consent was obtained from all participants before data collection. Collected data did not include personally identifiable information (e.g., name, ID number). The study followed the ethical principles of the Declaration of Helsinki (1995 version, revised in Edinburgh in 2000), ensuring respect for autonomy, confidentiality, and non-maleficence. Participants were fully informed about the study’s purpose, procedures, potential benefits, and risks, and they voluntarily chose to participate. They could withdraw at any time without explanation. All data were securely stored on password-protected computers and were accessible only to the research team.

### 2.5 Statistical analysis

Data were entered into EpiData 3.1 by two researchers and analyzed using SPSS 27.0. Normally distributed measurement data are presented as mean ± SD, while non-normally distributed data are shown as median (*Q*_1_, *Q*_3_). Group comparisons were conducted using independent *t*-tests or Mann-Whitney *U*-tests, as appropriate. Categorical variables are reported as frequencies and percentages (%), with group differences analyzed by chi-square (χ^2^) tests. Spearman correlation analysis was used to assess associations between physical, cognitive, and social frailty scores and sarcopenia indicators. Multivariate analysis was performed using binary logistic regression. Two-tailed tests were applied, and *P* < 0.05 was considered statistically significant.

## 3 Result

### 3.1 Participant characteristics

A total of 325 MHD patients were included, of whom 66.8% were male, with ages ranging from 24 to 91 years. Most participants (59.7%) had an education level of elementary school or below. Regarding lifestyle factors, 4.0% were current drinkers and 8.9% were current smokers.

Based on the Fried Frailty Phenotype, Mini-Mental State Examination (MMSE), and Social Frailty Scale, participants were categorized into non-coexisting frailty (*n* = 289) and coexisting frailty groups (*n* = 36). The prevalence of coexisting physical, cognitive, and social frailty was 11.1%. Regarding single-domain frailty, 91 (28.0%) had physical frailty, 64 (19.7%) had cognitive frailty, and 86 (26.5%) had social frailty. For two-domain frailty, 36 (11.1%) had both physical and cognitive frailty, 44 (13.5%) had cognitive and social frailty, and 24 (7.4%) had physical and social frailty.

Significant differences between the coexisting and non-coexisting frailty groups were observed for age, educational level, place of residence, BMI, calf circumference, mid-upper arm circumference, mid-arm muscle circumference, phase angle, extracellular water ratio, body cell mass index, dialyzer type, vascular access site, serum creatinine, potassium, phosphorus, albumin, prealbumin, parathyroid hormone, triglycerides, LDL-C, Subjective Global Assessment (SGA), social support, International Physical Activity Questionnaire (IPAQ) scores, depression scores, and presence of sarcopenia (*P* < 0.05). Detailed comparisons are presented in Table 1.

**TABLE 1:** Univariate analysis of general characteristics and coexistence of physical, cognitive, and social frailty among patients undergoing maintenance hemodialysis (*n* = 325).

Variable	Total sample (*n* = 325)	Non-coexisting frailty (*n* = 289)	Coexisting frailty (*n* = 36)	Statistical value	*P*
Age, years	61.00 (51.00, 71.00)	59.00 (49.00, 68.00)	78.00 (71.25, 83.00)	7.660^2)^	< 0.001
Sex [*n* (%)]				0.151^3)^	0.697
Male	217 (66.8)	194 (67.1)	23 (63.9)		
Female	108 (33.2)	95 (32.9)	13 (26.1)		
Education level [*n* (%)]				9.273^3)^	0.055
Illiterate	33 (10.2)	29 (10.1)	4 (11.1)		
Primary school	82 (25.2)	66 (22.8)	16 (44.5)		
Junior high school	112 (34.5)	103 (35.6)	9 (25.0)		
Vocational/high school	60 (18.5)	57 (19.7)	3 (8.3)		
College and above	38 (11.7)	34 (11.8)	4 (11.1)		
Residence				4.798^3)^	0.028
Rural	251 (77.2)	218 (75.4)	33 (91.7)		
Urban	74 (22.8)	71 (24.6)	3 (8.3)		
Smoking history [*n* (%)]				4.242^3)^	0.120
Never smoker	283 (87.1)	248 (85.8)	35 (97.2)		
Current smoker	29 (8.9)	29 (10.0)	0 (0.0)		
Former smoker	13 (4.0)	12 (4.2)	1 (2.8)		
Alcohol consumption history [*n* (%)]				1.712^3)^	0.425
Never drinker	303 (92.9)	267 (92.4)	35 (97.2)		
Current drinker	13 (4.0)	13 (4.5)	0 (0.0)		
Former drinker	10 (3.1)	9 (3.1)	1 (2.8)		
Primary cause of ESRD [*n* (%)]				5.742^3)^	0.332
Chronic glomerulonephritis	156 (48.0)	140 (48.4)	16 (44.4)		
Diabetic nephropathy	90 (27.7)	81 (28.1)	9 (25.0)		
Hypertensive nephropathy	21 (6.5)	16 (5.5)	5 (13.9)		
Polycystic kidney disease	22 (6.8)	20 (6.9)	2 (5.6)		
IgA nephropathy	11 (3.4)	11 (3.8)	0 (0.0)		
Others	25 (7.7)	21 (7.3)	4 (11.1)		
Diabetes comorbidity [*n* (%)]				0.263^3)^	0.608
With diabetes	204 (62.8)	180 (62.3)	24 (66.7)		
Without diabetes	121 (37.2)	109 (37.7)	12 (33.3)		
BMI	22.25 (19.00, 24.75)	22.50 (19.60, 24.80)	19.20 (17.05, 22.20)	−3.253^2)^	< 0.001
Calf circumference	31.05 ± 3.65	31.43 ± 3.44	28.00 ± 3.85	5.565^1)^	< 0.001
Mid-upper arm circumference	26.20 (23.65, 28.70)	26.50 (24.10, 28.80)	23.35 (21.75, 26.15)	−3.860^2)^	< 0.001
Mid-arm muscle circumference	22.10 (20.20, 24.00)	22.30 (20.30, 24.35)	20.05 (18.10, 22.15)	−4.083^2)^	< 0.001
Phase angle	5.42 (4.49, 6.58)	5.58 (4.76, 6.70)	3.92 (3.03, 4.45)	−6.395^2)^	< 0.001
Extracellular water ratio	0.390 (0.380, 0.401)	0.389 (0.379, 0.398)	0.411 (0.395, 0.419)	5.697^2)^	< 0.001
Body cell mass index	10.99 ± 2.08	11.16 ± 2.08	9.67 ± 1.52	5.277^1)^	< 0.001
Body fat percentage	18.40 (10.30, 25.50)	18.15 (10.40, 25.20)	19.10 (9.20, 26.80)	0.136^2)^	0.892
Dialysis vintage	44.00 (16.50, 79.00)	44.00 (17.00, 81.50)	43.50 (14.50, 72.50)	−0.579^2)^	0.562
Dialyzer type [*n* (%)]				4.003^3)^	0.045
Low-flux dialyzer	82 (25.2)	68 (23.5)	14 (38.9)		
High-flux dialyzer	243 (74.8)	221 (76.5)	22 (61.1)		
Vascular access site [*n* (%)]				14.278^3)^	0.003
Catheter	46 (14.2)	34 (11.8)	12 (33.3)		
Arteriovenous graft	10 (3.1)	9 (3.1)	1 (2.8)		
Left arm arteriovenous fistula	220 (67.7)	204 (70.6)	16 (44.4)		
Right arm arteriovenous fistula	49 (15.1)	42 (14.5)	7 (2.4)		
Urea	15.49 (8.90, 21.07)	15.81 (8.82, 21.07)	14.00 (11.50, 20.28)	0.112^2)^	0.911
Creatinine	773.09 ± 293.16	793.46 ± 289.89	609.54 ± 270.48	3.615^1)^	< 0.001
Fasting blood glucose	6.42 (4.96, 8.23)	6.45 (4.99, 8.26)	6.05 (4.30, 7.80)	−0.916^2)^	0.360
Potassium	4.75 ± 0.67	4.78 ± 0.66	4.50 ± 0.70	2.359^1)^	0.001
Calcium	2.21 ± 0.21	2.22 ± 0.20	2.09 ± 0.27	0.670^1)^	0.503
Magnesium	1.01 ± 0.17	1.01 ± 0.17	0.96 ± 0.14	1.604^1)^	0.110
Phosphorus	1.65 ± 0.53	1.67 ± 0.52	1.43 ± 0.54	2.605^1)^	0.010
Total bilirubin	9.70 (7.90, 11.90)	9.60 (7.90, 11.80)	10.20 (7.95, 12.25)	0.754^2)^	0.451
Albumin	38.90 (36.50, 41.10)	39.30 (36.80, 41.30)	35.85 (33.65, 38.45)	−5.173^2)^	< 0.001
Globulin	27.83 ± 4.20	27.74 ± 4.15	28.54 ± 4.59	−1.076^1)^	0.283
Prealbumin	0.29 ± 0.09	0.30 ± 0.08	0.24 ± 0.10	3.709^1)^	< 0.001
Hemoglobin	113.00 (102.00, 123.00)	113.50 (103.00, 122.00)	112.00 (96.50, 126.00)	−0.212^2)^	0.832
Neutrophil-to-lymphocyte ratio	3.90 (2.90, 5.30)	4.00 (3.10, 5.30)	3.20 (2.35, 5.75)	−0.315^2)^	0.753
Parathyroid hormone (PTH)	188.50 (81.00, 320.35)	196.75 (90.35, 333.15)	96.3 (41.75, 206.28)	−3.161^2)^	0.002
Vitamin D	22.44 (14.99, 30.66)	22.47 (14.86, 30.31)	22.46 (15.60, 34.82)	0.591^2)^	0.555
β_2_-microglobulin	31.50 (23.46, 41.25)	31.32 (23.46, 40.71)	34.99 (22.31, 43.46)	0.457^2)^	0.648
hs-CRP	3.40 (1.30, 8.39)	3.10 (1.30, 8.20)	5.35 (1.35, 8.87)	0.842^2)^	0.400
Triglycerides	1.43 (1.02, 2.22)	1.58 (1.03, 2.24)	1.21 (0.75, 1.66)	−2.640^2)^	0.008
Total cholesterol	3.46 ± 1.00	3.50 ± 0.97	3.15 ± 1.16	1.970^1)^	0.050
HDL-C	0.85 (0.72, 1.03)	0.87 (0.72, 1.02)	0.85 (0.71, 1.14)	0.300^2)^	0.764
LDL-C	1.57 (1.19, 2.02)	1.63 (1.22, 2.03)	1.39 (0.96, 1.79)	−2.498^2)^	0.012
Kt/V	1.42 ± 0.29	1.39 ± 0.29	1.42 ± 0.34	0.026^1)^	0.979
SGA	10.00 (9.00, 12.00)	9.00 (8.00, 10.00)	13.50 (12.00, 16.50)	7.736^2)^	< 0.001
International physical activity	396.00 (0.00, 693.00)	495.00 (0.00, 803.50)	0.00 (0.00, 280.50)	−4.196^2)^	<0.001
Sarcopenia [*n* (%)]				77.327^3)^	< 0.001
With sarcopenia	52 (16.0)	28 (4.4)	24 (53.8)		
Without sarcopenia	273 (84.0)	261 (95.6)	13 (46.2)		
Depression	47.00 (35.00, 55.00)	46.00 (35.00, 53.00)	54.00 (47.50, 66.00)	3.734^2)^	< 0.001
Social support	26.00 (21.00, 31.00)	26.00 (22.00, 32.00)	22.00 (19.00, 24.50)	−4.374^2)^	< 0.001

1), t statistic; 2), Mann-Whitney U-test; 3) chi-square statistic.

### 3.2 Multivariate analysis of factors associated with coexisting frailty

Binary logistic regression was performed with coexisting physical, cognitive, and social frailty as the dependent variable and variables with statistical significance in univariate analysis as independent variables (coding methods in Table 2). The results indicated that sarcopenia (OR = 8.018, 95% CI 2.479∼25.932, *P* < 0.001), age (OR = 1.152, 95% CI 1.084∼1.224, *P* < 0.001), prealbumin level (OR = 0.002, 95% CI 0.000∼0.501, *P* = 0.028), social support (OR = 0.887, 95% CI 0.798∼0.985, *P* = 0.025), and MQSGA score (OR = 1.300, 95% CI 1.096∼1.541, *P* = 0.003) were identified as influencing factors (Table 3).

**TABLE 2:** The assignment of independent variables.

Variable	Coding method
Gender	Male = 1, female = 2
Education level	Illiterate = 1; primary school = 2; junior high school = 3; high school = 4; bachelor’s degree or above = 5
Dialyzer type	Low-flux dialyzer = 1; high-flux dialyzer = 2
Vascular access	Catheter = 1; synthetic graft = 2; left upper limb autogenous arteriovenous fistula = 3; right upper limb arteriovenous fistula = 4

**TABLE 3:** Binary logistic regression analysis of factors influencing coexistence of physical, cognitive, and social frailty in maintenance hemodialysis patients (*n* = 325).

Variable	β	Standard error	OR	95% CI	*P*-value
Age	0.142	0.031	1.152	1.084∼1.224	< 0.001
Prealbumin	−6.408	2.916	0.002	0.000∼0.501	0.028
Sarcopenia	2.082	0.599	8.018	2.479∼25.932	< 0.001
Social support	−0.120	0.054	0.887	0.798∼0.985	0.025
MQSGA score	0.262	0.087	1.300	1.096∼1.541	0.003

### 3.3 Correlation analysis between dimensions of physical, cognitive, and social frailty coexistence, and sarcopenia, diagnostic parameters

Spearman correlation analysis showed that the subdomains of physical, cognitive, and social frailty coexistence were all significantly correlated with sarcopenia diagnostic indicators (grip strength, skeletal muscle mass index, and 6 m walking speed) (*P* < 0.01) (Table 4).

**TABLE 4:** Correlation analysis between physical, cognitive, and social frailty dimensions and sarcopenia diagnostic indicators.

Variable	Sarcopenia	Skeletal muscle index	6 m walking speed	Grip strength
Physical frailty	0.390**	−0.300**	0.590**	−0.551**
Cognitive frailty	−0.309**	0.202**	−0.463**	0.339**
Social frailty	0.355**	−0.356**	0.555**	−0.363**
Coexisting frailty	0.488**	−0.275**	0.440**	−0.318**

**Indicates *P* < 0.01.

### 3.4 Cross-sectional associations between sarcopenia and frailty dimensions

In the crude analysis, sarcopenia was strongly associated with physical frailty (OR = 11.267, 95% CI 5.695–22.289), cognitive frailty (OR = 7.681, 95% CI 4.017–14.686), social frailty (OR = 10.035, 95% CI: 5.166–19.493), and the coexistence of physical, cognitive, and social frailty (OR = 18.643, 95% CI 8.418–41.285) (*P* < 0.001). Notably, the prevalence of physical, cognitive, and social frailty was 28%, 19.7%, and 26.5%, respectively, while 11.1% of patients exhibited the coexistence of all three dimensions. After stepwise adjustment for potential confounders, including demographic characteristics, lifestyle factors, and disease-related variables, these associations remained statistically significant (*P* < 0.001). Detailed results are presented in Table 5.

**TABLE 5:** Cross-sectional associations of sarcopenia with coexisting physical, cognitive, and social frailty, as well as with each individual dimension. in different models.

Frailty type	Prevalence (%)	Model 1		Model 2		Model3		Model 4	
		OR (95% CI)	*P*	OR (95% CI)	*P*	OR (95% CI)	*P*	OR (95% CI)	*P*
Physical frailty	28%	11.267 (5.695∼22.289)	< 0.001	9.507 (4.544∼18.891)	< 0.001	6.253 (2.829∼13.819)	< 0.001	6.347 (2.861∼14.082)	< 0.001
Cognitive frailty	19.7%	7.681 (4.017∼14.686)	0.020	6.535 (2.773∼15.400)	< 0.001	7.095 (2.795∼18.010)	< 0.001	7.471 (2.914∼19.154)	< 0.001
Social frailty	26.5%	10.035 (5.166∼19.493)	< 0.001	9.649 (4.273∼21.790)	< 0.001	5.936 (2.513∼14.024)	< 0.001	6.057 (2.543∼14.427)	< 0.001
Coexisting frailty	11.1%	18.643 (8.418∼41.285)	< 0.001	24.341 (7.704∼76.905)	< 0.001	21.582 (6.431∼72.432)	< 0.001	24.563 (7.010∼86.077)	< 0.001

Molel 1: unadjusted. Model 2: adjusted for demographic factors (age, gender, education, residence). Model 3: further adjusted for lifestyle factors (smoking, alcohol, BMI). Model 4: further adjusted for disease-related factors (primary disease, diabetes, dialysis duration).

## 4 Discussion

### 4.1 Coexistence of physical, cognitive, and social frailty at a moderate level among patients undergoing maintenance hemodialysis

The results of this study revealed that among 325 patients receiving maintenance hemodialysis (MHD), the proportions of individuals exhibiting frailty in 0, 1, 2, and all 3 domains were 60.0% (*n* = 195), 15.4% (*n* = 50), 32.0% (*n* = 104), and 11.1% (*n* = 36), respectively. These findings indicate a moderate level of coexisting multidimensional frailty, which is lower than the prevalence reported in a related study by Imamura et al. (15), where 15.4% of 344 elderly MHD patients exhibited frailty in all three domains. This discrepancy may be attributed to differences in the age composition of the study populations. While the present study included adult MHD patients aged ≥ 18 years, Imamura et al.’s (15) study focused solely on elderly individuals, who are generally more susceptible to frailty across multiple dimensions. Furthermore, our results suggest that overlaps exist among different frailty domains in MHD patients. Overlap between cognitive and social frailty was relatively common (13.5%), whereas the co-occurrence of physical and social frailty was less frequent (7.4%), which is notably lower than the 23.8% reported by Zhou et al. (30). This difference may be partially explained by variations in the residential settings of the participants. In our study, a high proportion of patients (77.2%) resided in rural areas, where individuals are more likely to engage in traditional agricultural labor, maintain higher levels of daily physical activity, and participate in more frequent social interactions. These lifestyle factors may help delay the onset of both physical and social frailty.

### 4.2 Coexistence of physical, cognitive, and social frailty in maintenance hemodialysis patients is influenced by multiple factors

#### 4.2.1 Advanced age as a risk factor for the coexistence of physical, cognitive, and social frailty in MHD patients

The findings of this study indicate that increasing age is associated with a higher risk of concurrent physical, cognitive, and social frailty among patients undergoing maintenance hemodialysis (MHD). From a cognitive perspective, aging leads to gradual degeneration of multiple organ systems and structural changes in the brain, such as reduced synaptic density and alterations in presynaptic active zones (31), resulting in varying degrees of damage across different brain regions and subsequent cognitive decline. Physiologically, aging is also accompanied by progressive deterioration of physical function. In MHD patients, this decline is exacerbated by dialysis-related protein loss and toxin accumulation, contributing to the gradual loss of skeletal muscle mass and strength. Moreover, chronic inflammation and immunosenescence compromise the body’s immune defense mechanisms, increasing the risk of complications such as pulmonary infections and dialysis access-related bloodstream infections, which further aggravate physical frailty (32). These complications, including malnutrition and infection, often limit patients’ mobility and reduce their engagement in social activities, thereby elevating their risk of social frailty. Given these risks, healthcare professionals should prioritize comprehensive frailty screening in elderly MHD patients and develop individualized intervention strategies tailored to their specific conditions, needs, and lifestyle preferences. Community centers and dialysis facilities can organize regular cognitively stimulating group activities-such as board games, handicrafts, and other interactive programs-to slow neurodegenerative processes, improve cognitive function, and promote social engagement. For elderly MHD patients receiving home-based care, participation in online social networking, virtual shopping, or telehealth consultations-with the support of family members or community staff-can help meet their psychosocial needs and strengthen their social connections.

#### 4.2.2 Prealbumin and SGA scores as influencing factors for the coexistence of physical, cognitive, and social frailty in maintenance hemodialysis patient

As important tools for assessing malnutrition and inflammatory status, prealbumin levels and Subjective Global Assessment (SGA) scores together reflect the nutritional risk in patients undergoing maintenance hemodialysis (MHD). Prealbumin, a negative acute-phase protein with a short half-life, serves as an early indicator of impaired protein synthesis and increased skeletal muscle catabolism when its levels decline (33). The SGA scale, by comprehensively evaluating body weight changes, dietary intake, physical activity, and functional capacity, provides a global assessment of a patient’s nutritional status. Malnutrition is a common complication in MHD patients and is closely associated with adverse outcomes, frequently coexisting with frailty (34). The present study found that higher prealbumin levels and lower SGA scores (indicating better nutritional status) were protective factors against the coexistence of physical, cognitive, and social frailty in MHD patients. In terms of physical frailty, prolonged malnutrition prompts the body to preferentially utilize fat reserves to preserve muscle mass. However, approximately 10% of energy still needs to be supplied by amino acids from muscle tissue to the brain and glycolytic organs, resulting in progressive muscle mass loss and impaired physical function, ultimately contributing to physical frailty (35). Furthermore, Mori and Shanely (36) suggested that skeletal muscle wasting represents a shared pathophysiological mechanism underlying both frailty and malnutrition. Nutrient loss during dialysis and disturbances in mineral metabolism further accelerate muscle degradation. Since muscle function typically declines before structural changes occur, reduced handgrip strength-an important indicator of muscle function-signals an elevated risk of physical frailty. In the cognitive domain, a cross-sectional study (37) reported that chronic malnutrition impairs neuronal regeneration, disrupts neurotransmitter balance, and damages brain structures, thereby contributing to multidimensional cognitive decline, including deficits in visuospatial learning, executive function, complex reasoning, short-term memory, and reaction stability. In the social domain, dialysis-related malnutrition is often accompanied by fatigue, low mood, and depression. As health deteriorates, patients experience a progressive loss of physical function, limiting their capacity for daily activities and reducing their willingness to engage in social participation. Consequently, they may voluntarily or involuntarily withdraw from social interactions (38). Moreover, malnutrition increases the risk of hospitalization and healthcare costs (39), further intensifying the financial burden. This may lead patients to deliberately reduce contact with others and restrict social activities, exacerbating social frailty. Therefore, healthcare providers should conduct routine nutritional screening and collaborate with dietitians to develop individualized nutritional management plans that consider the needs of both patients and their primary caregivers. On the basis of nutritional improvement, it is also essential to promote physical exercise and cognitive training, and to encourage patients to reestablish social connections to improve their overall frailty status.

#### 4.2.3 Social support as a protective factor against the coexistence of physical, cognitive, and social frailty in maintenance hemodialysis patients

Social support refers to the assistance and encouragement that individuals receive from family members, relatives, friends, colleagues, and other social entities, encompassing material resources, emotional support, and informational aid (40). The results of this study demonstrate that social support serves as a protective factor against the coexistence of physical, cognitive, and social frailty in patients undergoing maintenance hemodialysis, which aligns with findings from previous studies (41, 42). In the context of physical frailty, Cao (43) found that MHD patients with high levels of social support can fully leverage both objective and perceived resources from their social networks. These resources facilitate access to positive emotional experiences, health knowledge, and medical services, which collectively enhance self-care capacity, promote behavioral change, and alleviate physical and psychological symptoms-ultimately mitigating physical frailty. Additionally, studies have shown that a low level of perceived social support is significantly associated with anxiety and depression among MHD patients (44). Depression, in turn, may influence gastrointestinal motility via neurohumoral pathways, impairing nutrient intake and absorption. This can result in reduced physical endurance and muscle strength, thereby increasing the risk of physical frailty (45). Regarding cognitive frailty, the chronic nature of kidney disease and the lifestyle changes associated with dialysis often necessitate adaptation to altered social roles and functions. A low level of social support may hinder patients’ ability to mobilize available resources, reduce motivation to cope with health challenges, and decrease participation in cognitively stimulating activities. This may accelerate brain cell aging and cognitive decline, ultimately impairing cognitive function (46). In the domain of social frailty, a robust social support system helps alleviate negative emotions such as loneliness and hopelessness, enhances coping efficacy and confidence in treatment, and fosters a sense of social identity and emotional fulfillment. These effects collectively promote individual wellbeing and the restoration of social functioning, thereby reducing the risk of social frailty (42, 47). Therefore, healthcare providers should actively work to establish and improve comprehensive social support systems while encouraging patients to rebuild their social networks and fully utilize available social resources and coping capabilities. Patients should be supported in participating in shared decision-making and self-management of their health. Additionally, the formation of interdisciplinary medical teams-comprising general practitioners, dialysis specialists, and community-based dialysis nurses-can provide patients and their primary caregivers with professional knowledge and skills. Complementary support interventions such as respite care services, psychological counseling, and peer-family support groups tailored to specific diseases can further enhance support at the professional, familial, and peer levels. This multidimensional “hospital–home–community” integrated support model can help prevent or delay the onset of physical, cognitive, and social frailty, ultimately improving the quality of life for MHD patients (43, 48, 49).

### 4.3 Association between sarcopenia and the coexistence of physical, cognitive, and social frailty in maintenance hemodialysis patients

The correlation analysis in this study revealed a significant positive association between sarcopenia and the coexistence of physical, cognitive, and social frailty in maintenance hemodialysis (MHD) patients (*r* = 0.488, *P* < 0.001), suggesting that increasing severity of sarcopenia may be associated with a higher risk of multidimensional frailty. Furthermore, binary logistic regression analysis identified sarcopenia as an independent risk factor for frailty coexistence (OR = 8.018, *P* < 0.001), indicating that MHD patients with sarcopenia have approximately eight times the risk of developing coexisting physical, cognitive, and social frailty compared to those without sarcopenia.

#### 4.3.1 Positive correlation between physical frailty and sarcopenia in maintenance hemodialysis patients

The results of the correlation analysis indicated a significant positive association between physical frailty and sarcopenia in maintenance hemodialysis (MHD) patients (*r* = 0.390, *P* < 0.001), suggesting that greater severity of physical frailty is associated with an increased risk of sarcopenia. However, previous research (50) has shown that frailty may increase the risk of sarcopenia and serve as a significant predictor of its onset. The causal relationship between the two conditions remains unclear. Metsemakers et al. (19) proposed that sarcopenia may act as a precursor syndrome to frailty, which is driven by declining physiological reserves and multisystem impairments, including the musculoskeletal, endocrine, and immune systems. Frailty primarily emphasizes the decline in overall physical function, whereas sarcopenia focuses on the loss of muscle mass and function. The significant correlation observed in this study may reflect shared pathophysiological mechanisms. Nutrient losses during dialysis and inadequate intake due to cardiovascular and gastrointestinal complications can result in protein–energy wasting, leading to impaired muscle protein synthesis and increased catabolism. This, in turn, contributes to progressive skeletal muscle deterioration and ultimately to frailty (51), In addition, inflammatory cytokines may serve as a common pathophysiological mechanism for both conditions. Inflammatory mediators can activate the nuclear factor-κB signaling pathway, suppress myocyte differentiation, and promote muscle atrophy (52), eventually progressing to frailty. Therefore, healthcare professionals should consider early identification of sarcopenia using tools such as the SARC-Calf scale or dynamic risk prediction models. Personalized intervention plans should be developed based on the patient’s needs, incorporating nutritional supplementation, physical exercise, and psychological support. For patients with elevated inflammatory markers, pharmacological interventions and non-pharmacological strategies-such as anti-inflammatory diets and structured physical activity-should be implemented to reduce inflammation and slow the progression of sarcopenia and frailty.

#### 4.3.2 Negative correlation between cognitive frailty and sarcopenia in maintenance hemodialysis patients

Previous studies (53, 54) have indicated that sarcopenia increases the risk of cognitive impairment, potentially due to a decline in patients’ ability to perform activities of daily living and diminished cognitive and executive capacity for engaging in health-promoting behaviors, such as regular exercise, adequate nutrition, and self-management. In the present study, correlation analysis revealed that among the diagnostic indicators of sarcopenia, cognitive function was significantly associated with muscle strength, muscle mass, and physical performance, with stronger correlations observed for muscle strength and physical performance. These findings are consistent with those reported by Chen et al. (55). Handgrip strength tests and gait assessments require coordination of hand movements, postural control, and gait stability, which are dependent on higher-order cognitive functions such as executive function, attention, and visuospatial abilities (56). Moreover, gait speed has been recognized by the International Academy on Nutrition and Aging as the “sixth vital sign” (57), and its decline has been identified as a significant predictor of cognitive deterioration (58). Therefore, in clinical practice, healthcare providers are advised to routinely screen for cognitive impairment in patients exhibiting reduced muscle strength or slower gait speed, and to implement targeted interventions to improve limb strength. A quasi-experimental study conducted in Shanghai demonstrated that a multicomponent exercise program combined with cognitive training significantly improved sarcopenia-related indicators in elderly patients with comorbid psychiatric disorders, with the most pronounced improvement observed in muscle strength, followed by gait speed (59). Accordingly, prior to prescribing exercise programs, clinicians should assess patients’ individual functional status and stratify them appropriately. Exercise regimens should be personalized based on patient preferences and daily routines and should incorporate multimodal components-such as aerobic, balance, and resistance training-to effectively enhance both muscle function and cognitive capacity.

#### 4.3.3 Positive correlation between sarcopenia and social frailty in maintenance hemodialysis patients

The correlation analysis in this study revealed a significant association between social frailty and diagnostic indicators of sarcopenia, particularly handgrip strength and physical performance, which is consistent with previous findings (60). One possible explanation is that patients with arteriovenous fistulas tend to limit the use and weight-bearing of the fistula arm to preserve vascular access, leading to progressive decline in grip strength and restriction in daily functions such as lifting. Additionally, as dialysis vintage increases, sedentary behavior becomes more prevalent among MHD patients, resulting in reduced gait speed, diminished self-care ability, and loss of functional independence. This functional decline may prompt patients to intentionally reduce the size of their social networks and avoid forming new social connections, instead maintaining interactions only with familiar network members (61). Furthermore, this study found that gait speed was more strongly correlated with social frailty than handgrip strength, suggesting that social frailty may be more closely associated with hypokinetic (low-mobility) sarcopenia. In clinical practice, attention should be given to patients with impaired gait speed, and targeted functional training programs should be implemented to improve muscle endurance, mobility, and balance. Enhancing patients’ physical independence may play a critical role in preventing or mitigating social frailty in the MHD population.

### 4.4 Strengths and limitations of the study

The innovative aspects of our study are as follows: Firstly, the measures of coexisting physical frailty, cognitive frailty, social frailty and sarcopenia were widely used and validated tools to understand the questions thoroughly. Secondly, this is one of the very few studies that evaluate the effect of sarcopenia, coexistence of physical, cognitive and social frailty on MHD patients which suggests improving the quality of life and developing public health strategies.

Nevertheless, several limitations of this study should be acknowledged. First, there may be a bidirectional association between the coexistence of physical, cognitive, and social frailty and sarcopenia; however, due to its cross-sectional design, this study was unable to establish causal relationships. Second, depressive symptoms, social support, and social frailty were assessed using self-reported questionnaires, which may be subject to recall bias or misinterpretation. Third, cognitive function was evaluated using the MMSE, chosen for its practicality, yet its sensitivity in distinguishing normal cognition from mild cognitive impairment is limited.

Moreover, although frailty is particularly prevalent among older patients with chronic kidney disease, age-stratified analyses were not conducted in this study. This decision was primarily due to limitations in sample size, as further stratification could have resulted in insufficient statistical power within certain subgroups. In addition, socioeconomic variables such as household income, occupation, and access to healthcare were not systematically collected, which restricted our ability to assess the influence of economic status on frailty and sarcopenia. Future studies are encouraged to incorporate comprehensive socioeconomic measures and perform age-based subgroup analyses to enhance understanding of multidimensional frailty across diverse patient populations.

## 5 Conclusion

This study investigated the influencing factors of coexisting physical, cognitive, and social frailty in MHD patients. The results demonstrated that advanced age, prealbumin levels, SGA scores, social support levels, and sarcopenia were significant determinants of this multidimensional frailty syndrome, these results provide clinicians with a reference for identifying high-risk MHD patients and give public health policymakers a scientific approach to taking targeted interventions. Furthermore, This research further explore the two-way relationship between coexistence of physical, cognitive and social frailty and sarcopenia. Spearman correlation analysis revealed a significant positive association between the coexistence of physical, cognitive, and social frailty and sarcopenia. In clinical practice, healthcare providers should prioritize sarcopenia risk screening in MHD patients and implement multimodal interventions combining aerobic/resistance exercise prescriptions with nutritional support to reduce sarcopenia incidence.

## Data Availability

The raw data supporting the conclusions of this article will be made available by the authors, without undue reservation.
